# Supplementary material for: Identification of potent high-affinity secondary nucleation inhibitors of Aβ42 aggregation from an ultra-large chemical library using deep docking
Source: Mol Syst Biol. 2025 Nov 5;22(1):5. doi: 10.1038/s44320-025-00159-5 (PMC12759071; doi:10.1038/s44320-025-00159-5)
Supplement: Supplementary file 1 — Appendix [file 44320_2025_159_MOESM1_ESM.pdf]

Appendix

Identification of potent high-affinity secondary nucleation inhibitors of Aβ42 aggregation from an ultra-large chemical library using Deep Docking

Michaela Brezinova[1], Z. Faidon Brotzakis[1], Robert I. Horne[1], Vaidehi Roy Chowdhury[1], Rebecca C. Gregory[1], Yuqi Bian[1], Alicia Gonzalez-Diaz[1], Francesco Gentile[2,3], and Michele Vendruscolo*[1]

[1]Centre for Misfolding Diseases, Yusuf Hamied Department of Chemistry, University of Cambridge, 12 Union Rd, Cambridge, CB2 1EZ, United Kingdom
[2]Department of Chemistry and Biomolecular Sciences, University of Ottawa, 10 Marie Curie Pvt, Ottawa, ON K1N 6N5, Ontario, Canada
[3]Ottawa Institute of Systems Biology, University of Ottawa, 451 Smyth Road, Ottawa, ON K1H 8M5, Ontario, Canada

Contents

List of Appendix Figures

Appendix Figure S1 — AKTA chromatogram of size exclusion chromatography (SEC) for Aβ42 2

List of Appendix Tables

Appendix Table S1 — Final selection of 59 molecules for experimental validation. 3
Appendix Table S2 — Results of experimental validation of the tested 35 molecules and the reference hit. 5

*Corresponding author: mv245@cam.ac.uk

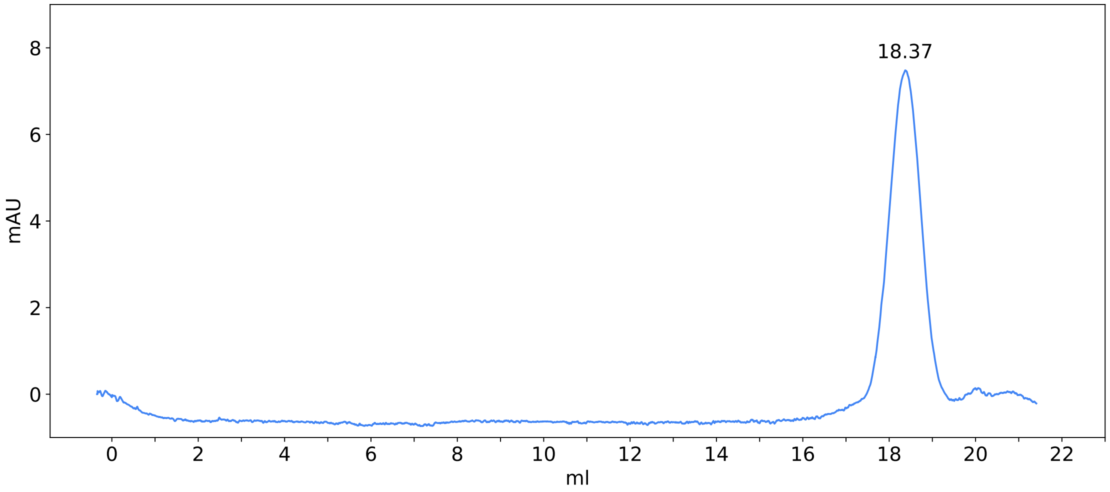

Appendix Figure S1: **AKTA chromatogram of size exclusion chromatography (SEC) for Aβ42**. The chromatogram confirms the presence and successful isolation of monomers.

Appendix Table S1: Final selection of 59 molecules for experimental validation.

Name	MPO score	DeePred-BBB pass	VINA score	FRED score
ZINC000921188341	1.0	Yes	-7.3	-7.1
ZINC000754688989	1.0	Yes	-7.4	-7.7
ZINC001540268885	1.0	Yes	-7.7	-6.2
ZINC001216568302	1.0	Yes	-7.4	-8.0
ZINC001247605921	1.0	Yes	-7.9	-5.8
ZINC001248286470	1.0	Yes	-7.2	-7.7
ZINC000770802518	1.0	Yes	-7.8	-7.5
ZINC001241792879	1.0	Yes	-7.5	-7.5
ZINC001206018071	0.99	Yes	-7.7	-6.8
ZINC001534462639	0.98	Yes	-7.3	-7.9
ZINC001126369666	0.98	Yes	-7.4	-7.2
ZINC001360850146	0.98	Yes	-7.4	-7.0
ZINC001206017809	0.98	Yes	-7.3	-8.1
ZINC001217702527	0.98	Yes	-7.4	-7.5
ZINC001165231142	0.98	Yes	-7.4	-6.8
ZINC001458129326	0.98	Yes	-7.4	-8.1
ZINC001243732531	0.98	Yes	-7.4	-8.0
ZINC001469428141	0.98	Yes	-7.7	-6.5
ZINC000002605557	0.98	Yes	-7.7	-6.2
ZINC001128686159	0.98	Yes	-8.0	-7.2
ZINC001217629271	0.98	Yes	-7.7	-6.1
ZINC001148948653	0.98	Yes	-7.6	-7.0
ZINC001530228439	0.98	Yes	-7.7	-6.5
ZINC001212771256	0.98	Yes	-7.3	-7.1
ZINC000897222660	0.98	Yes	-7.9	-5.7
ZINC000000527929	0.98	Yes	-7.6	-6.4
ZINC000170637172	0.98	Yes	-7.2	-8.3
ZINC001247689327	0.98	Yes	-7.6	-7.6
ZINC001520365977	0.98	Yes	-7.5	-7.4
ZINC000105712195	0.98	Yes	-7.8	-5.8
ZINC000002412961	0.98	Yes	-7.7	-6.5
ZINC001678712262	0.98	Yes	-7.7	-6.4
ZINC000303649646	0.98	Yes	-7.7	-7.2
ZINC001584221852	0.98	Yes	-7.5	-6.5
ZINC000078729682	0.98	Yes	-7.9	-6.1
ZINC000730201302	0.98	Yes	-8.1	-6.5
ZINC001639603977	0.98	Yes	-7.4	-7.4
ZINC001128043262	0.97	Yes	-7.5	-7.3
ZINC001564046727	0.96	Yes	-7.9	-7.1
ZINC001356606705	0.96	Yes	-7.3	-7.3

Continued on next page

Name	MPO score	DeePred-BBB pass	VINA score	FRED score
ZINC001538634391	0.95	Yes	-7.3	-7.8
ZINC001458911787	0.92	Yes	-7.7	-6.1
ZINC000460665332	0.92	Yes	-7.4	-7.0
ZINC001164143635	0.92	Yes	-7.4	-7.5
ZINC001320245585	0.92	Yes	-7.3	-7.0
ZINC000643141156	0.92	Yes	-7.5	-6.7
ZINC001469626916	0.92	Yes	-8.0	-6.0
ZINC001449473922	0.92	Yes	-7.8	-5.9
ZINC000735459325	0.92	Yes	-7.7	-6.9
ZINC001186023151	0.92	Yes	-7.7	-6.9
ZINC000582893105	0.92	Yes	-7.4	-7.6
ZINC000579127806	0.92	Yes	-7.4	-6.9
ZINC001680912672	0.92	Yes	-7.5	-6.8
ZINC000605388160	0.92	Yes	-7.5	-6.6
ZINC001475901927	0.92	Yes	-7.4	-6.8
ZINC000914531369	0.92	Yes	-7.4	-6.8
ZINC000047827947	0.92	Yes	-7.5	-6.5
ZINC000047827944	0.92	Yes	-7.5	-6.7
ZINC000481533573	0.91	Yes	-7.6	-7.7

Appendix Table S2: Results of experimental validation of the tested 35 molecules and the reference hit.

Label	Name	SMILES	Norm $t_{1/2}$	Hit
M1	ZINC001678712262	O=C(NCCc1ccc(-c2ccccc2)cc1)N1Cc2ccccc2CO1	9.01	Yes
M2	ZINC000643141156	O=C(NCC1CCCc2ccccc21)c1ccc(-c2ccccc2)[nH]c1=O	1.62	Yes
M3	ZINC001564046727	CC1(c2ccccc2)CCN(C(=O)NCC2Cc3ccccc3C2)CC1	1.47	No
M4	ZINC001680912672	Cc1cccc(C2=CCN(C(=O)NCc3ccc4[nH]c(C)cc4c3)CC2)c1	2.71	Yes
M5	ZINC001639603977	Cc1nc2c(c(NCCc3ccc(-c4ccccc4)cc3)n1)CCCC2	0.97	No
M6	ZINC001128043262	Cc1cccc2c1CN(C(=O)COc1ccc(-c3ccccc3)cc1)CC2	1.44	No
M7	ZINC001530228439	Cc1cccc(-c2ccc(CNC(=O)C3Cc4cccc(C)c4O3)cc2)c1	2.34	Yes
M8	ZINC000460665332	CC1(C)C2CN(C(=O)CCCc3ccc4c(c3)-c3ccccc3C4)CC21	1.62	Yes
M9	ZINC000303649646	Cc1cccc(-c2ccc(CNC(=O)c3ccc4c(c3)nnn4C)cc2)c1	1.28	No
M10	ZINC001584221852	Cc1cccc2c1OCC(NC(=O)Cc1ccc(-c3ccccc3)cc1)C2	2.78	Yes
M11	ZINC000730201302	Cc1cn(-c2ccccc2)nc1NC(=O)c1ccc(-c2ccccc2)cc1	6.92	Yes
M12	ZINC000078729677	O=C(CC1OCCc2ccccc21)Nc1ccc2c(c1)Cc1ccccc1-2	3.76	Yes
M13	ZINC001475901927	O=C(NCc1ncccn1)Nc1ccc2c(c1)Cc1ccccc1-2	1.11	No
M14	ZINC001540268885	CCc1ccc2c(c1)CCN(C(=O)C1(C)Cc3ccccc3C(=O)O1)C2	0.0	No
M15	ZINC000770802518	Cc1cccc(C2=CCN(C(=O)Cc3cnn(-c4ccccc4)n3)CC2)c1	3.3	Yes
M16	ZINC001360850146	Cc1cccc(-c2ccc(CNC(=O)c3cc4n(n3)CCCC4=O)cc2)c1	0.88	No
M17	ZINC001449473922	O=C(Nc1nccc2ccccc12)c1ccc(-c2ccccc2)[nH]c1=O	1.35	No
M18	ZINC001356606705	CC1(CNC(=O)Cc2ccc(-c3ccccc3)cc2)Cc2ccccc2C1	3.33	Yes
M19	ZINC001458129326	Cc1cc(C)nc(NC(=O)COc2ccc(-c3ccccc3)cc2)c1	2.74	Yes
M20	ZINC001126369666	Cc1cccc(-c2ccc(CNC(=O)c3cc4n(n3)CCCC4)cc2)c1	1.3	No

Continued on next page

Label	Name	SMILES	Norm $t_{1/2}$	Hit
M21	ZINC001534462639	CC(C)c1cccc(C(=O)NCCc2ccc(-c3ccccc3)cc2)n1	1.72	Yes
M22	ZINC001320245585	Cc1cc(C)nc(NC(=O)NC2CCCN(C(=O)c3ccccc3)C2)c1	1.16	No
M23	ZINC001458911787	Cc1cccc2c1CN(C(=O)Cc1ccc(-c3ccccc3)cc1)CC2	2.87	Yes
M24	ZINC000105712195	Cc1nc(-c2ccccc2)ccc1C(=O)Nc1ccn(-c2ccccc2)n1	3.76	Yes
M25	ZINC000921188341	Cc1cc(Oc2ccc3c(c2)[nH]c2ccccc23)nc(-c2cccnc2)n1	2.74	Yes
M26	ZINC000047827944	CC(NCc1ccc(-c2ccccc2)cc1)c1ccc2c(c1)CCC(=O)N2	1.28	No
M27	ZINC000897222660	Cc1cccc([C@@H]2C[C@H]2c2nc(-c3cc4c([nH]c3=O)CCCC4)no2)c1	1.13	No
M28	ZINC001248286470	c1ccc(-c2ccc(CCc3nc(-c4cccnc4)no3)cc2)cc1	1.82	Yes
M29	ZINC001538634391	Cc1cc(C)cc(CCNC(=O)c2ccc(-c3ccccc3)cc2)c1	5.68	Yes
M30	ZINC000754688989	CN1C(=O)CCc2cc(C(=O)C=Cc3ccc(-c4ccccc4)o3)ccc21	2.36	Yes
M31	ZINC001128686159	Cn1nc(-c2ccccc2)nc1NC(=O)c1ccc(-c2ccccc2)cc1	3.67	Yes
M32	ZINC000579127806	O=C(NC1CC=C(c2ccc(F)cc2)CC1)c1cc(=O)[nH]c(C2CC2)n1	1.09	No
M33	ZINC000735459325	Cc1cccc(-c2ccc(CNC(=O)c3cc4c([nH]c3=O)CCC4)cc2)c1	1.23	No
M34	ZINC000170637172	Cc1cc(N)nc(SCC(=O)c2ccc3c(c2)Cc2ccccc2-3)n1	1.27	No
M35	ZINC000481533573	Cc1nnc(-c2ccc(C(=O)Nc3nc(-c4ccccc4)nn3C)cc2)[nH]1	1.35	No
adapalene	ZINC000003784182	COc1ccc(-c2ccc3cc(C(=O)O)ccc3c2)cc1C12CC3CC(CC(C3)C1)C2	5.22	Reference Hit
