# Supplementary material for: Identification of potent high-affinity secondary nucleation inhibitors of Aβ42 aggregation from an ultra-large chemical library using deep docking
Source: Mol Syst Biol. 2025 Nov 5;22(1):5. doi: 10.1038/s44320-025-00159-5 (PMC12759071; doi:10.1038/s44320-025-00159-5)
Supplement: Supplementary file 6 — Source data Fig. 4 [file 44320_2025_159_MOESM6_ESM.zip › SD Figure 4/4B/Supporting data from instrument for Fig 4A.pdf]

File Properties

Evaluation File

Name:	20240407_CM3_FC4-3_MCK_Ab42-ligand_M11-M1-analyte.bme
Path:	C:\Bia Users\Vaidehi vr358
Size:	6 487 040

User Information

Performed By:	Administrator
Current User:	Administrator

Created With Software

Name:	Biacore T200 Evaluation Software
Version:	1.0

Notebook

Result

Result File

Name:	20240407_CM3_FC4-3_MCK_Ab42-ligand_M11-M1-analyte.blr
Path:	C:\Bia Users\Vaidehi vr358
Size:	6 479 872 bytes

Run Information

Type:	Method Builder
Method:	C:\Bia Users\Methods And Templates\Vaidehi vr358\20240407_CM3_FC4-3_MCK_Ab42-fibrils-ligand_M11-M1-analyte.Method
Cycles:	45
Start:	4/7/2024 9:35:33 PM
End:	4/8/2024 8:12:15 AM

Instrument

Instrument Type:	BiacoreT200
Instrument Id:	1646191
IFC:	TYPE105

User Information

Run Performed By:	Administrator

Created With Software

Name:	Biacore T200 Control Software
Version:	1.0

Chip Information

Chip Id:	240327: Vaidehi Ab42 fib
Chip Lot No:	10347728
Chip Name:	CM3
First Dock Date:	3/27/2024 4:08:39 PM
Last Modification Date:	3/27/2024 4:08:39 PM
Last Use Date:	4/6/2024 8:12:37 PM

Immobilization in Fc=1

Immobilization Date:
Immobilization Result:
Ligand:
Final Response [RU]:

Immobilization in Fc=2

Immobilization Date:
Immobilization Result:
Ligand:
Final Response [RU]:

Immobilization in Fc=3

Immobilization Date:
Immobilization Result:
Ligand:

File Properties (continued)

Final Response [RU]:

Immobilization in Fc=4
Immobilization Date:
Immobilization Result:
Ligand:
Final Response [RU]:

Notebook

Affinity: 'M11', fit: '1. Steady State Affinity'

Curve: Fc=4-3 Ligand: N/A Sample: M11 Temp: 25°C

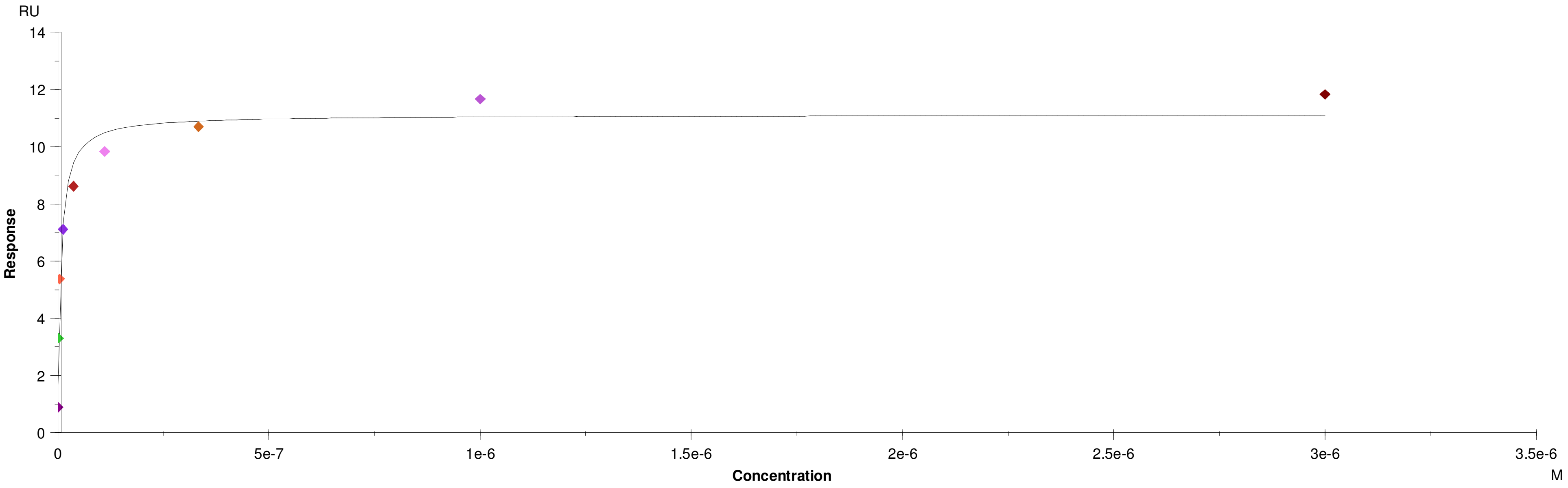

Report table

KD (M)	Rmax (RU)	offset (RU)	Chi² (RU²)
7.502E-9			0.613
	10.01	1.103	

Parameters table

KD (M)	SE(KD)	Rmax (RU)	SE(Rmax)	offset (RU)	SE(offset)
7.502E-9	2.6E-9				
		10.0	0.82	1.1	0.80
