# Supplementary material for: Identification of potent high-affinity secondary nucleation inhibitors of Aβ42 aggregation from an ultra-large chemical library using deep docking
Source: Mol Syst Biol. 2025 Nov 5;22(1):5. doi: 10.1038/s44320-025-00159-5 (PMC12759071; doi:10.1038/s44320-025-00159-5)
Supplement: Supplementary file 6 — Source data Fig. 4 [file 44320_2025_159_MOESM6_ESM.zip › SD Figure 4/4A/Supporting data from instrument for Fig 4A.pdf]

File Properties

Evaluation File

Name:	N/A

User Information

Performed By:	Administrator
Current User:	Administrator

Created With Software

Name:	Biacore T200 Evaluation Software
Version:	1.0

Notebook

Result

Result File

Name:	20240425_CM3_FC2-1_MCK_Ab42-fibril-ligand_M1-analyte.blr
Path:	C:\Bia Users\Vaidehi vr358
Size:	2 961 408 bytes

Run Information

Type:	Method Builder
Method:	C:\Bia Users\Methods And Templates\Vaidehi vr358\20240425_CM3_FC2-1_MCK_Ab42-fibrils-ligand_M1-analyte.Method
Cycles:	22
Start:	4/25/2024 1:20:33 PM
End:	4/25/2024 6:11:23 PM

Instrument

Instrument Type:	BiacoreT200
Instrument Id:	1646191
IFC:	TYPE105

User Information

Run Performed By:	Administrator

Created With Software

Name:	Biacore T200 Control Software
Version:	1.0

Chip Information

Chip Id:	240423: VRC CM3 Ab42 fib
Chip Lot No:	10347728
Chip Name:	CM3
First Dock Date:	4/23/2024 6:20:58 PM
Last Modification Date:	4/23/2024 6:20:58 PM
Last Use Date:	4/23/2024 6:22:22 PM

Immobilization in Fc=1

Immobilization Date:
Immobilization Result:
Ligand:
Final Response [RU]:

Immobilization in Fc=2

Immobilization Date:
Immobilization Result:
Ligand:
Final Response [RU]:

Immobilization in Fc=3

Immobilization Date:
Immobilization Result:
Ligand:
Final Response [RU]:

File Properties (continued)

Immobilization in Fc=4
Immobilization Date:
Immobilization Result:
Ligand:
Final Response [RU]:

Notebook

Affinity: ‘M1’, fit: ‘1. Steady State Affinity’

Curve: Fc=2-1 Ligand: N/A Sample: M1 Temp: 25°C

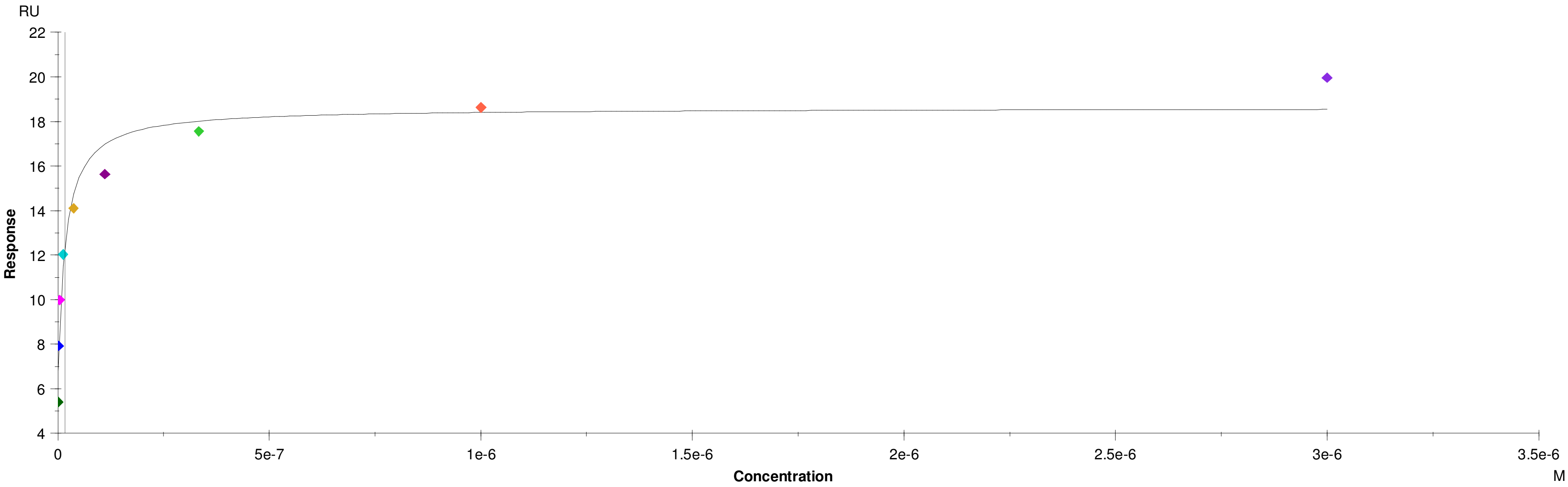

Report table

KD (M)	Rmax (RU)	offset (RU)	Chi² (RU²)
1.739E-8			1.39
	12.14	6.480	

Parameters table

KD (M)	SE(KD)	Rmax (RU)	SE(Rmax)	offset (RU)	SE(offset)
1.739E-8	6.9E-9				
		12.1	1.1	6.5	0.94
