# Supplementary material for: Identification of potent high-affinity secondary nucleation inhibitors of Aβ42 aggregation from an ultra-large chemical library using deep docking
Source: Mol Syst Biol. 2025 Nov 5;22(1):5. doi: 10.1038/s44320-025-00159-5 (PMC12759071; doi:10.1038/s44320-025-00159-5)
Supplement: Supplementary file 9 — Expanded View Figures [file 44320_2025_159_MOESM9_ESM.pdf]

Expanded View Figures

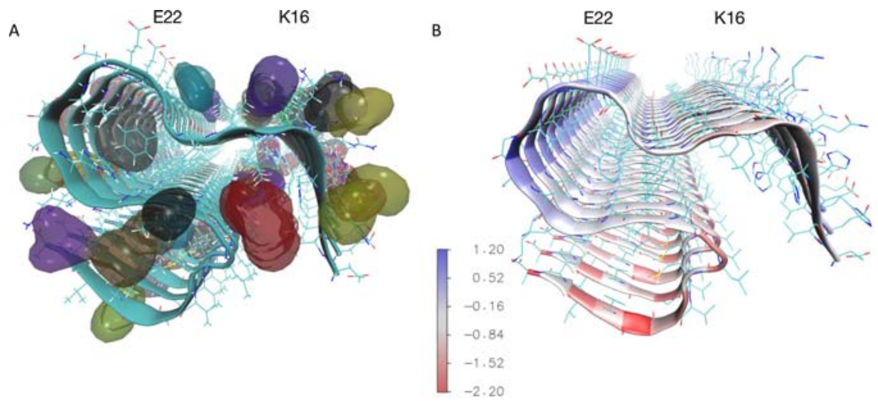

Figure EV1. Selection of the binding site on the amyloid fibril structure of Aβ42.

The structure of the amyloid fibril of Aβ42 used in this work (PDB 2MXU) is depicted in a cartoon and line representation. (**A**) Prediction of binding sites using Fpocket (Le Guilloux et al, 2009), colored with different volumes. The selected binding site comprises residues ^{16}KVFAHLE^{22}. (**B**) Solubility predictions scores per residue from CamSol (Sormanni et al, 2015). The color code ranges from red, corresponding to low solubility, to blue corresponding to high solubility. The selected binding site involves residues of low to medium solubility.

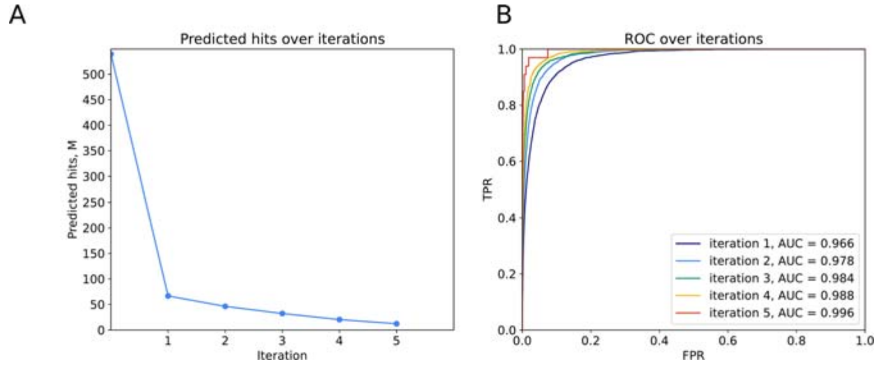

Figure EV2. Iterative implementation of the deep docking pipeline.

(**A**) Number of molecules (in millions, M) predicted to be hits as a function of the iteration. In the iterative process, we reduced the initial library size of 539 million to 12 million predicted hits. (**B**) ROC curves and increasing AUC values over iterations (FPR false-positive rate, TPR true-positive rate)

 © The Author(s)

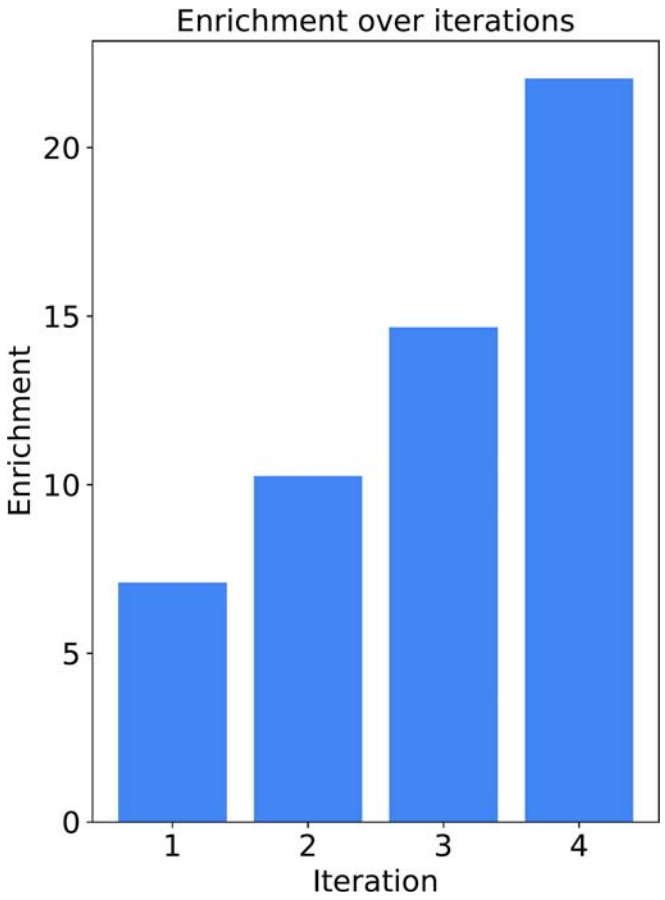

Figure EV3. The enrichment of a random sample of model-predicted hits increases with iterations.

The enrichment for iteration n was calculated using $E_n = \frac{TP_{n,n}}{TP_{n,0}}$, where $TP_{n,n}$ are virtual true positives in a random sample of predicted hits generated by the best model of iteration n (in our case, this random sample is the sample used to enrich the training set for iteration $n+1$) given model-established hit threshold of iteration n. $TP_{n,0}$ represent virtual true positives in the initial random sample of the original library (in our case, the training set of the first iteration), given the model-established hit threshold of iteration n. The virtual true positives in both cases mean molecules of which the Vina score passed the respective threshold.

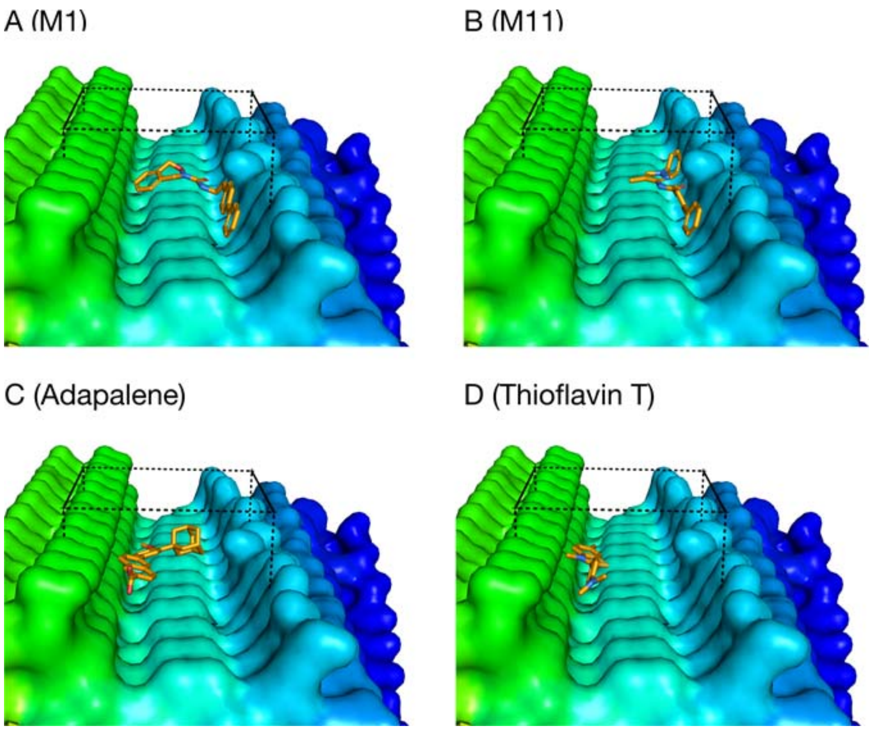

Figure EV4. Predicted best docking poses of the compounds discussed in this work to Aβ42 fibrils within the binding site (black dashed line).

(**A**, **B**) M1 (**A**) and M11 (**B**) are the two most potent hits. (**C**, **D**) Predicted docking poses for adapalene (**C**) and thioflavin T (**D**) are also shown for comparison. Panels are created using PyMOL (Schrödinger LLC, 2021).

 © The Author(s)

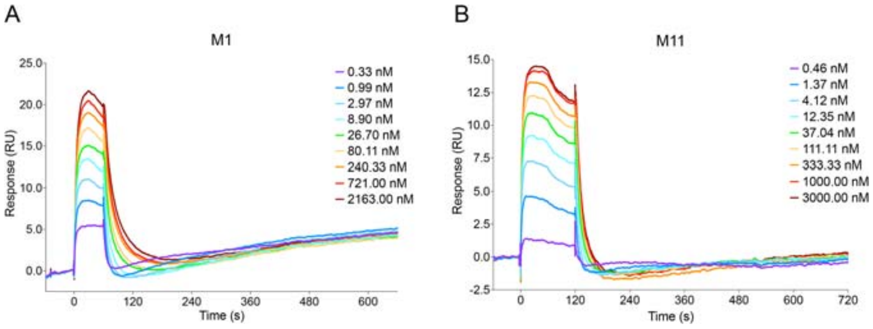

Figure EV5. SPR sensorgram of binding kinetics of drug molecules to Aβ42 fibrils.

The SPR response of binding of M1 (**A**) or M11 (**B**) at different concentrations to Aβ42 fibrils immobilized at 1700–1800 RU onto CM3 sensor chip (Cytiva) are shown after removal of outlier replicates ($n = 2$).
